# Decytabine enhances cytotoxicity induced by oxaliplatin and 5-fluorouracil in the colorectal cancer cell line Colo-205

**DOI:** 10.1186/1475-2867-9-10

**Published:** 2009-04-27

**Authors:** Sylwia Flis, Agnieszka Gnyszka, Irena Misiewicz-Krzemińska, Jacek Spławiński

**Affiliations:** 1 Department of Pharmacology, National Medicines Institute, Warsaw, Poland; 2 Confocal Microscopy Laboratory, Department of Cell Biology, National Medicines Institute, Warsaw, Poland

## Abstract

**Background:**

DNA methylation is an epigenetic phenomenon known to play an important role in the development of cancers, including colorectal cancer (CRC). Aberrant methylation of promoter regions of genes is potentially reversible, and if methylation is important for cancer survival, demethylation should do the opposite. To test this we have addressed the hypothesis that DNA methyltransferase inhibitors (DNMTi), decytabine and zebularine, potentiate inhibitory effects of classical anti-CRC cytostatics, oxaliplatin and 5-fluorouracil (5-FU), on survival of CRC cells *in vitro*.

**Results:**

Isobole and median effect analysis revealed that decytabine shows potent synergistic interaction with oxaliplatin and 5-FU and that this is probably not the class effect of DNMTi as zebularine shows strong antagonistic interaction with oxaliplatin. The synergistic combination treatment was also applied to the cultures to investigate their mechanisms of action. We have shown that combinations of decytabine with cytostatics produced dose-dependent growth inhibition and treatment-induced apoptosis.

**Conclusion:**

The observed synergism between decytabine and cytostatics is most probably related to the augmented apoptotic signal and allowed for significant (both biologically and statistically) reduction of the cytotoxic doses of cytostatics used.

## Background

Altered patterns of 5-cytosine methylation at CpG islands located in the promoter regions of genes are implicated in the development of a wide range of human cancers. This change in DNA methylation may cause the transcriptional silencing of important cancer-controlling genes such as tumor suppressors and caretaker genes. Examples include genes encoding: *RB *in retinoblastoma [1]; *VHL *in renal carcinoma [2]; *p15 *in gliomas and leukemias [3]; *BRCA1 *in breast cancer [4]; *E-cadherin *in hepatocellular carcinoma, breast cancer, and prostate cancer [5]; *GSTP1 *in prostate, breast, and renal cancer [6]; and *p16*^*INK*4*a *^in virtually all human cancers studied including colorectal carcinoma (CRC) [7]. In colorectal carcinoma (CRC) aberrant DNA methylation may be linked to the causal mechanism in colon carcinogenesis [8]. Recently, it was reported that aberrant methylation of promoter regions of genes as *p15*, *p16*^*INK*4*a*^, estrogen receptor, *MLH1 *and *APC *– all probably involved in the development of CRC is potentially reversible [9] and therefore may constitute the target for demethylating agents. Therefore, reversal of methylation by demethylating agents should lead to the inhibition of cancer. If this hypothesis is correct such agents should inhibit the survival of CRC cells *in vitro*. However, it seems pointless to study the effects of demethylating agents alone without combination with 5-fluorouracil (5-FU) and/or oxaliplatin that are used for CRC in the clinics as these cytostatics represent the backbone of the treatment of patients with CRC.

The question arises, therefore, whether the effect of combined treatment (demethylating agents with cytostatics) is superior to the treatment with each agent alone. To address this question we evaluated the effects of demethylating agents, decytabine and zebularine, in combination with cytostatics, oxaliplatin and 5-FU, on growth of cells of Colo-205 human CRC cell line. The aim of the study was to find out whether combinations of studied agents produced additive, antagonistic or synergistic interaction and in this way to set the stage for testing the drug combinations in *in vivo *conditions. The obtained results indicate that decytabine (but not zebularine) induced potent synergistic interaction with both studied cytostatics increasing their cytotoxicity at lower doses.

## Materials and methods

### Cell culture and drug treatment

As a model of colon cancer cells, the Colo-205 human colorectal cancer cell line, obtained from American Type Culture Collection (ATCC, Manassas, VA, USA) was used. The cells were cultured in RPMI 1640 medium (Gibco, Paisley, UK) supplemented with 5% (v/v) heat-inactivated fetal bovine serum (FBS, Gibco), 2 mM glutamax (Gibco), 100 units/ml penicillin, 100 μg/ml streptomycin and 250 ng/ml amphoterycin (Gibco) at 37°C in a humidified atmosphere including 5% CO_2_. Cells were incubated with drugs for 48 and 72 h. Both floating and attached cells were harvested for subsequent analysis.

### Drugs

The following drugs were studied: 5-fluorouracil (5-FU), oxaliplatin, zebularine, decytabine (Sigma, St. Louis, MI, USA). The concentrations of studied drugs were in the range from 1 to 200 μM. The drugs were dissolved in 100% dimethylsulfoxide (DMSO, Sigma) and then diluted in the media for experiments. The final concentration of DMSO, without effect on cell survival, was maintained at 0.2%. In all experiments control cells were incubated with DMSO.

### MTT assay

This assay relies on the ability of viable cells to metabolically reduce a yellow tetrazolium salt ([3-(4,5-dimethylthiazol-2-yl)-2,5-diphenyl tetrazolium bromide], MTT (Sigma) to purple formazan product via mitochondrial dehydrogenase activity. Cells were grown in 96-well plates (1 × 10^4 ^cells/200 μl/well). After incubation with the drugs, the medium was removed and the cells were treated with 50 μl of MTT for 4 h at 37°C. Next, 150 μl of solubilization solution (10% SDS) were added and the mixture was incubated at 37°C overnight. The solubilized formazan product was spectrophotometrically quantified using a microtiter plate reader, Power Wave XS (Bio-Tek, Winooski, VT, USA), at 570 nm wavelength.

### Drug interaction analysis

The nature of the interactions between studied drugs was analyzed with the help of izobologram [10] and median effect methods described by Chou and Talalay [11,12].

The Colo-205 cells were simultaneously incubated for 72 hours with combinations of either cytostatics (oxaliplatin or 5-FU) with decytabine or zebularine or with each agent alone. Isoboles were defined by effects of pair of studied drugs. The effects obtained by IC_50 _of either drug in the pair form the basis for the additivity line; synergism or antagonism was present when the same effect was obtained by the combination of drugs in lower or higher doses, respectively [10].

The CI method is a mathematical and quantitative evaluation of a two-drug pharmacologic interaction. Using data from the cytotoxicity experiments and CalcuSyn ver. 2.0 software (Biosoft, Cambridge, UK), CI values were generated over a range of fractional cell kill levels (Fa) from 0.05 to 0.95 (5–95% cell kill). CIs of <1 indicated synergism (the smaller the value, the greater the degree of synergy), CIs equal to 1 indicated additivity, and CIs>1 indicated antagonism. Data generated from the CI method were used to quantify the dose-reduction index (DRI) for the combination of two drugs. DRI represents the fold-decrease of each individual agent when two drugs are used in combination as opposed to alone to achieve a particular Fa. Each CI or DRI ratio represented here is the mean value derived from at least five independent experiments

### Flow cytometric analysis of cell cycle

Cells (~1 × 10^6^) were resuspended in 4 ml of 80% ethanol (- 20°C) and incubated at -20°C for 24 h, washed twice in phosphate-buffered saline (PBS), and stained with 50 μg/ml propidium iodide (PI) and 100 μg/ml RNase in 0.1% PBST solution (PBS supplemented with 0.1% Triton × 100) for 30 min in the dark at 4°C. The samples were then measured using a BD FACSCalibure flow cytometer (BD Biosciences, San Jose, CA, USA). The DNA histograms were analyzed using ModFit software (BD Biosciences, San Jose, CA, USA).

### Flow cytometric analysis of apoptosis

Apoptosis was measured, according to the manufacturer's instructions, using an annexin V-FITC kit (BD Biosciences, San Jose, CA, USA). The cells were collected after treatment, washed twice with PBS and centrifuged. The cell pellet was resuspended in ice-cold binding buffer. The annexin V-FITC and PI solutions were added to the cell suspension and mixed gently. The samples were then incubated for 15 min in the dark before flow cytometric analysis.

### Semi-quantitative RT-PCR

The mRNA levels of *CCNE1*, *ATM*, *p53*, *CASP3 *and *GAPDH *were analyzed by RT-PCR using total RNA from Colo-205 cells isolated using the Total RNA Kit (Kucharczyk TE, Warsaw, Poland), as described by the manufacturer. One hundred ng of total RNA was used in the reverse transcription reaction with Omniscript Reverse Transcriptase (Qiagen, Hilden, Germany) and oligo (dT)_18 _primer (Fermentas, Vilnius, Lithuania). The PCR amplifications were performed in a 50 μl total volume according to manufacture's instruction using HotStarTaq Master Mix (Qiagen), 3 μl of cDNA as a template and the following primers pairs: *CCNE1 *(5'-AACTCAACGTGCAAGCCTCG-3'; 5'-CATCTCCTGAACAAGCTCCA-3'), *ATM *(5'-GCCTT GAGTCTGTGTATTCG-3'; 5'-CCACTCAGAGACTCCACAGC-3'), *p53 *(5'-GCTCTGAC TGTACCACCATC-3'; 5'-CTTCTGACGCACACCTATTG-3'), *CASP3 *(5'-CTGGACTG TGGCATTGAGAC-3'; 5'-TGCAACTACCTGACTGGAAG-3') and *GAPDH *(5'-TCACCATC TTCCAGGAGCGA-3'; 5'-TGGTCATGAGTCCTTCCACG-3'). The *GAPDH *mRNA levels were used as internal controls. The amplified fragments were separated on 2% agarose gels, stained with ethidium bromide, photographed under UV light.

### Immunoblotting

The cells were washed with cold PBS buffer and then the nuclear and cytoplasmic fractions were extracted using the NE-PER extraction kit (Pierce, Rockford, IL, USA). Protein concentration in the samples was measured using BCA protein assay kit (Pierce, Rockford, IL, USA). Samples containing 30 μg of protein were denatured and fractionated by 12% SDS-PAGE. After electrophoresis, the proteins were transferred to a nitrocellulose membrane and probed with anti-human antibodies specific to: cyclin A1, D1 and PARP (Santa Cruz Biotechnology, Inc., Santa Cruz, CA, USA); p21, pro-caspase 8 (BD Biosciences, Pharmingen, Poland); pro-caspase 3, β-actin (Sigma, Poland). The signal was detected by a colorimetric method using the Opti-Amplified Immun-blot Assay kit (Bio-Rad, Hercules, CA, USA).

### Mitochondrial membrane potential (*ΔΨ*_*m*_) measurement

*ΔΨ*_*m *_was measured using MitoLight™ Apoptosis Detection Kit (Chemicon Int., Germany). Cells were treated with decytabine and cytostatics or their combinations for 48 h, stained according to the kit producer's protocol and examined by Olympus IX70 FV500 confocal microscope. The dye was excited with Ar-blue 488 nm laser. The fluorescence of monomer and aggregates of the dye were detected using 505–525 nm and 560–620 nm bandpass filters respectively. MitoLight (5,5',6,6'-tetrachloro-1,1',3,3'-tetraethylbenzimidazolylcarbo-cyanine chloride a mitochondrial dye included in the kit, stains mitochondria in living cells. In healthy cells, the dye accumulates in mitochondria, forming aggregates that emit red fluorescence, while in apoptotic cells the dye remains in cytoplasm and emits green fluorescence. [13].

### Statistical analysis

Data were presented as mean values ± SD (standard deviation). Statistical comparisons among groups were performed by Student's t-test or one-way analysis of variance (ANOVA) followed by Tukey post hoc test. Significance was assumed at p < 0.05 (marked with asterisks).

## Results

### Growth studies

**Figure 1:**
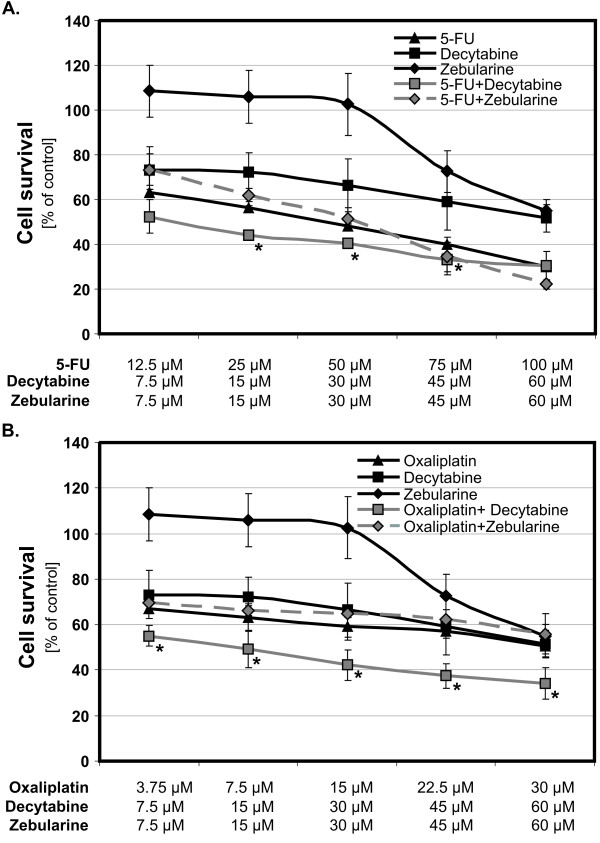
**Effect of combination treatment with cytostatics and DNMTi agents on survival of the Colo-205 cells**. Human colorectal cancer cells were exposed to 5-fluorouracil (5-FU) (**A**) or oxaliplatin (**B**) with and without demethylating agents, decytabine and zebularine (concentrations, in μM, below figures) for 72 h. Each bar represents the mean ± SD (n = 5), asterisks indicates significance at p < 0.05, for comparison with 5-FU or oxaliplatin.

The studied drugs, used in the concentration range from 3.75 to 100 μM, inhibited the survival of Colo-205 cells *in vitro*. Among tested compounds that were incubated with Colo-205 cells for 48 and 72 hours oxaliplatin and 5-FU showed the most potent inhibition of Colo-205 cells growth with the maximum following 72 hours incubation time (Fig. 1). The inhibitory effects of cytostatics were dose dependent and the correlation coefficients (estimated from the inhibitory dose-response curves) were above 0.9. Demethylating agents, decytabine and zebularine, have demonstrated different mode of inhibition: decytabine was already inhibitory at low concentrations (starting from 7.5 μM), whereas zebularine showed non-significant stimulation of Colo-205 cells growth at low concentration and was inhibitory, starting from concentrations of 45 μM.

The effects of various combinations of cytostatics with demethylating agents (decytabine and zebularine) on CRC cells survival are shown also in Fig. 1. Decytabine, from concentration of 7.5 μM up to 60 μM potentiated the inhibitory effects of oxaliplatin and in concentration from 7.5 μM to 30 μM potentiated the inhibitory effect of 5-FU on survival of CRC cells whereas zebularine, tested at concentrations up to 60 μM did not potentiate inhibitory effects of either cytostatic studied (Fig. 1).

### Interactions of cytostatics with decytabine or zebularine

**Figure 2:**
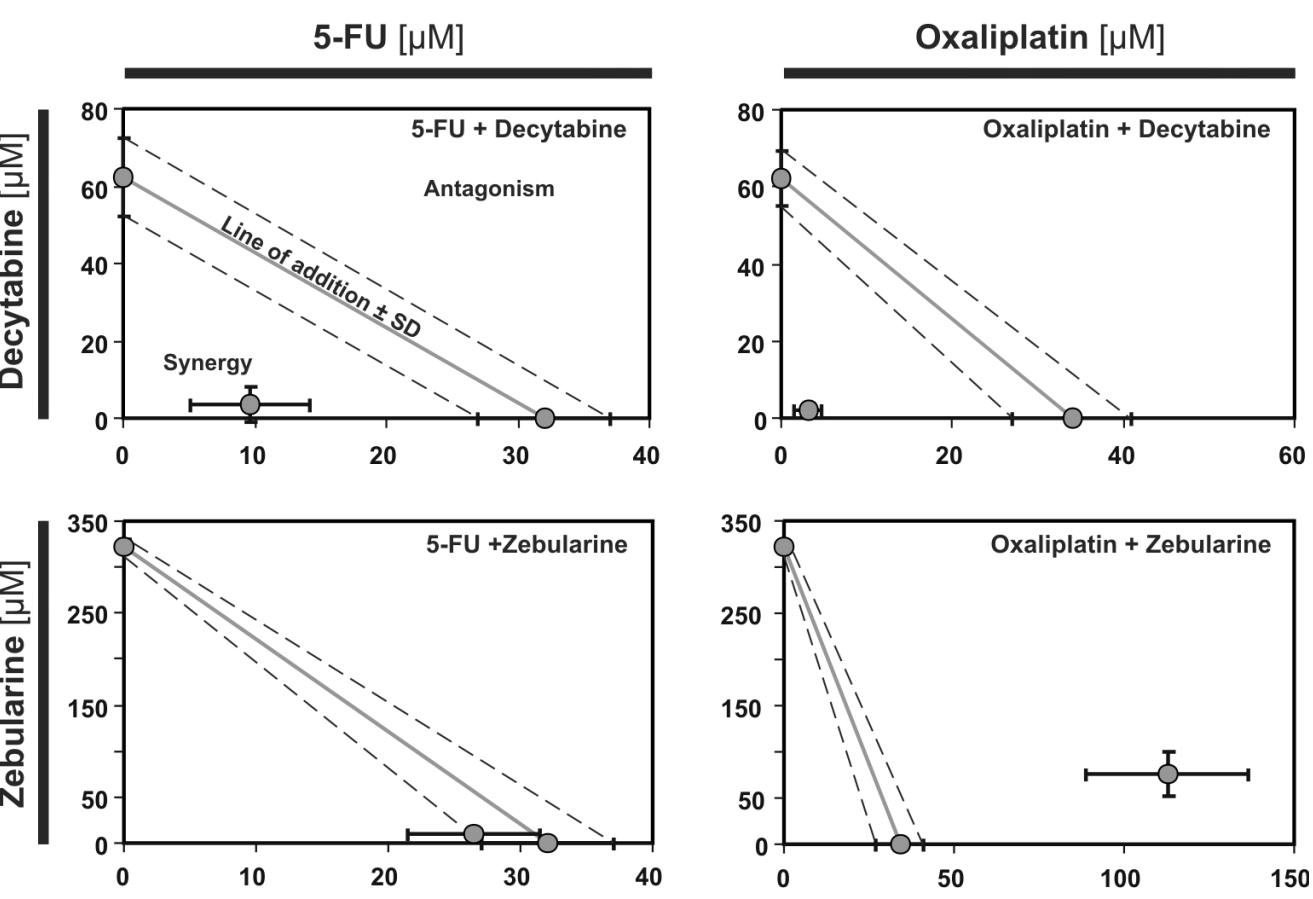
**Interaction of cytostatics with decytabine and zebularine**. Concentrations (μM) of particular drugs are indicated on x and y axis. The isobolograms were constructed by connecting the IC_50 _values of demethylating agents (on the ordinate) with the IC_50 _of oxaliplatin or 5-FU plotted on the abscissa. When the doses of two agents in combination are lower, or higher, than additive doses, the synergy or antagonism, respectively, is present.

The results of various combinations of decytabine and zebularine with cytostatics (oxaliplatin and 5-FU) on Colo-205 cells were analyzed with the help of isobolograms and median effects method of Chou and Talalay. Isobolograms indicated that interaction of decytabine with oxaliplatin or 5-FU resulted in strong synergistic interaction, whereas combination of zebularine with cytostatics produced different results; zebularine antagonized inhibitory effect of oxaliplatin on survival of CRC cells (Fig. 2) and was without influence (only additive interaction was observed) on effect of 5-FU.

Analysis performed with the help of median effect method (Table 1) revealed that combination of decytabine and cytostatics produced synergistic effects at all of three (25%, 50% and 75%) Fa levels achieving CI (Combination Index) values of ≤ 0.29 and ≤ 0.68 for oxaliplatin and 5-FU, respectively. Combination of the zebularine and cytostatics in the range of Fa ≥ 0.5 (50%) produced only antagonistic or additive effects.

**Table 1:** Combination index (CI) and dose reduction index (DRI) values for drugs combinations schedule after 72 h of simultaneous treatment.

**Drugs**		**Molar ratio**	**CI ± SD at Fraction affected level**			**DRI ± SD at Fraction affected level**					
			**25%**	**50%**	**75%**	**25%**		**50%**		**75%**	
				
**I**	**II**		**I+II**	**I+II**	**I+II**	**I**	**II**	**I**	**II**	**I**	**II**
**5-FU**	**Decytabine**	**1.7:1**	0.25 ± 0.1*	0.36 ± 0.1*	0.63 ± 0.3*	6.38 ± 1.9*	12.8 ± 5*	3.4 ± 0.7*	14.1 ± 3.1*	2.3 ± 0.6*	22.7 ± 9
	**Zebularine**	**1.7:1**	1.24 ± 0.2	0.76 ± 0.1*	0.68 ± 0.1	N/D	N/D	1.9 ± 0.7*	4.6 ± 1.8*	4.1 ± 2	2.2 ± 0.5*
**Oxaliplatin**	**Decytabine**	**1:2**	0.28 ± 0.1*	0.17 ± 0.1*	0.12 ± 0.1*	0.83 ± 0.4*	1.4 ± 0.4*	4.3 ± 0.43*	12.2 ± 3.1	N/A	N/A
	**Zebularine**	**1:2**	0.29 ± 0.3*	2.90 ± 0.5*	N/A	0.47 ± 0.3	1.3 ± 0.7	N/D	N/D	N/D	N/D

Data are expressed as means ± SD (n = 8 for CI; n ≥ 3 for DRI). *, *p *< 0.05; N/D, not determined; N/A, not achieved at the highest concentration tested. A CI value significantly less than 1 indicates synergy, a CI not significantly different from 1 indicates addition, and a CI significantly higher than 1 indicates antagonism. The DRI determines the magnitude of dose reduction allowed for each drug when given in synergistic combination, as compared with the concentration of a single agent that is needed to achieve the same effect. 25, 50 and 75% indicates selected values of fraction affected level.

The dose reduction index (DRI) indicated that the presence of decytabine allowed for 4.3-fold reduction of IC_50 _dose in the case of oxaliplatin and 3.4-fold reduction in the case of 5-FU (Table 1). The dose-reduction effects for combinations of zebularine with cytostatics were not evaluated because these combinations did not show synergistic effects.

### Effect of combined treatment with cytostatics and demethylating agents on cell cycle progression and induction of apoptosis

In the control culture, the percent of cells in each phase was stable. The tested compounds had exerted various effects on the cell cycle and apoptosis. Treatment with cytostatics resulted in a consistent increase of number of cells in the S phase at the expense of G1 phase. Decytabine alone arrested the cell cycle in the G2/M phase, whereas the effect of zebularine on CRC cell cycle was negligible.

**Figure 3:**
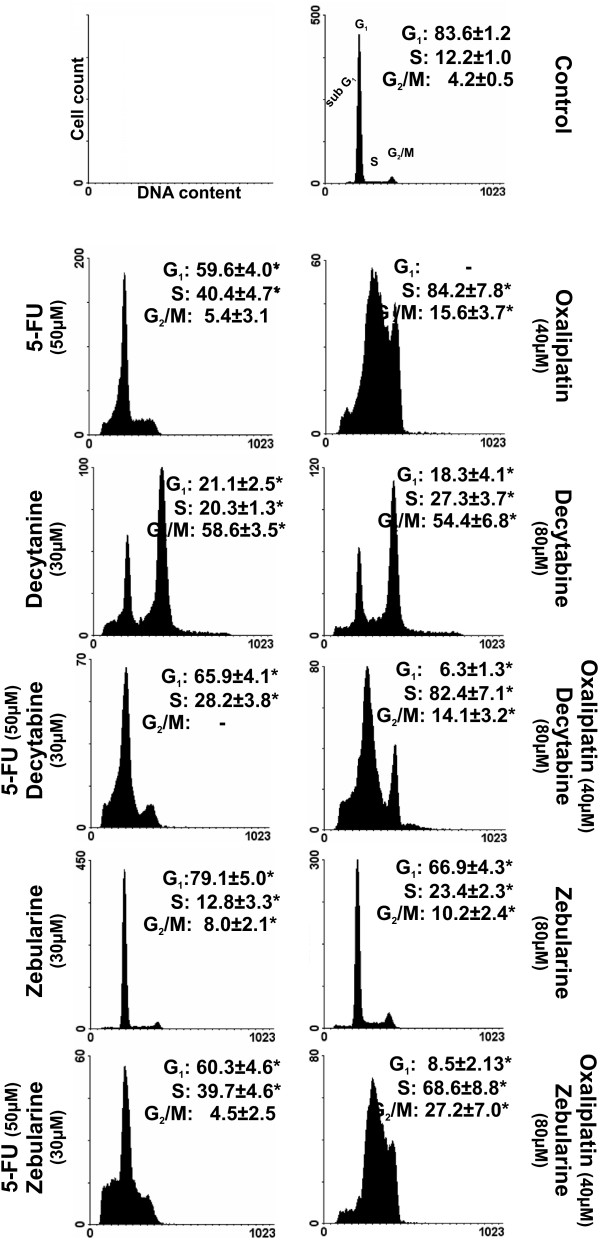
**Combinations of cytostatics with DNMTi agents induced changes in the cell cycle progression**. DNA histograms showing cell cycle phase distribution of Colo-205 cells treated with demethylating agents, cytostatics and their combinations (in concentrations as indicated) for 72 h and determined by FACS analysis, as described in Materials and Methods. Flow cytometric profiles of DNA content upon PI staining from a representative experiment are shown. Percentages are given for each cell phase as mean ± SD (n = 4). Asterisks indicates significance at p < 0.05, for comparison with control.

Combination of decytabine with oxaliplatin or 5-FU as compared with oxaliplatin or 5-FU alone, induced cell cycle arrest in the S phase and the G1/S phase, respectively (Fig. 3). Combination of zebularine with oxaliplatin, as compared to oxaliplatin alone, increased the number of cells in the G2/M phase and decreased the number of cells in the S phase (Fig. 3), whereas combination of zebularine with 5-FU exerted the same effect as 5-FU alone (Fig. 3).

**Figure 4:**
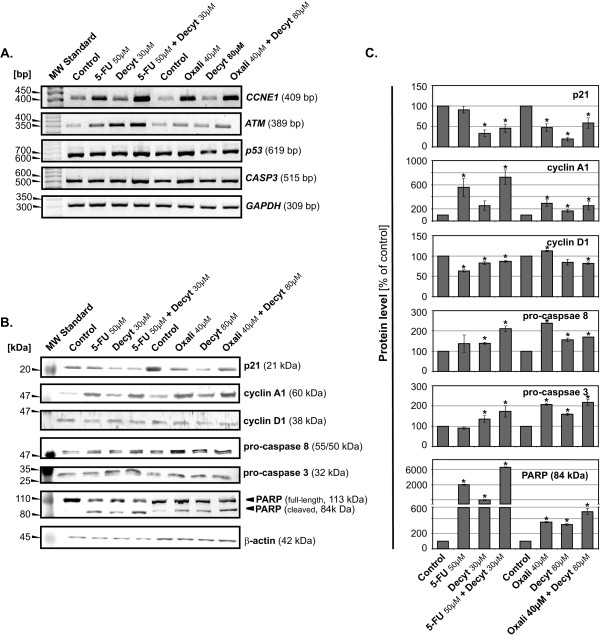
**Effect of combinations of cytostatics with decytabine on the cell cycle and apoptosis regulators**. (**A**), Analysis of *CCNE1*, *ATM*, *p53 *and *CASP3 *mRNA expression by semi-quantitative RT-PCR method after 72 h incubation of Colo-205 cells with cytostatics, decytabine and their combinations at concentrations as indicated. *GAPDH*, glyceraldehyde-3-phosphate dehydrogenase – internal control. (**B**), Immunoblotting analysis of the level of pro-apoptotic and cell cycle regulatory proteins following 72 h incubation of colorectal cells with cytostatics (5-FU, oxaliplatin), decytabine and their combinations at indicated concentrations. A representative blot of 2–3 ones for each particular protein is shown. The β-actin was used as a gel loading control. (**C**), Comparison of the relative amount of pro-apoptotic and cell cycle regulatory proteins, in samples shown in *panel B*, estimated by blot scanning and quantification of band intensities with the QuantiScan ver. 3.0 (Biosoft, UK) densitometry analysis software. The protein levels were normalized according to the gel loading control (β-actin) and presented as a percentage of control (level of particular protein in untreated cells). For particular protein, two to three blots were analyzed (n = 2 to 3). Each bar represents the mean ± SD; asterisks indicates significance at p < 0.05, for comparison with control.

Analysis of genes expression revealed that combination of decytabine with 5-FU, as compared with 5-FU alone, increased mRNA levels of: *CCNE1 *(a gene encoding cyclin E1), *ATM*, *p53*, and *CASP3 *(a gene encoding pro-caspase 3) (Fig. 4A). Analysis of proteins specific for cell cycle progression revealed that combination of decytabine with 5-FU, as compared with 5-FU alone, increased levels of cyclin A1 and decreased levels of cyclin D1 and p21 (Fig. 4B).

Analysis of genes expression revealed that combination of decytabine with oxaliplatin, as compared to oxaliplatin alone, increased mRNA level of *ATM*. Analysis of proteins specific for cell cycle progression revealed that combination of decytabine with oxaliplatin, as compared with oxaliplatin alone, increased level of cyclin A1 and decreased level of cyclin D1 (Fig. 4B).

**Figure 5:**
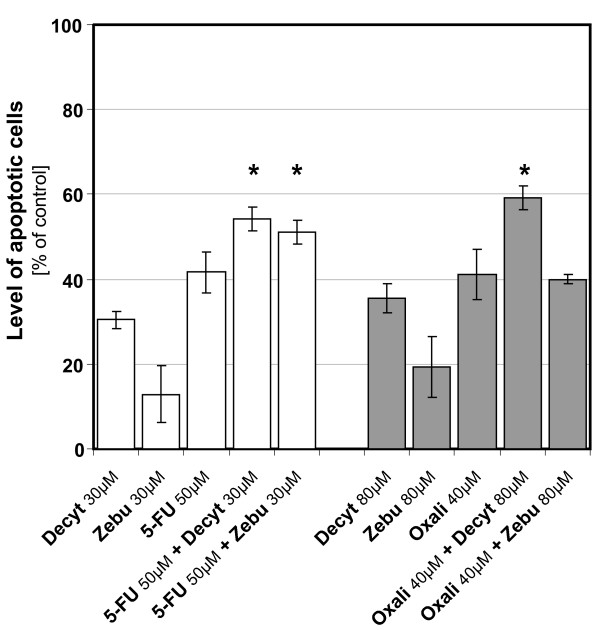
**Effect of cytostatics and DNMTi agents combinations on induction of apoptosis**. Percentage of apoptosis determined by FACS analysis, after 72 h treatment with demethylating agents, cytostatics and their combinations in concentrations as indicated. Each column represented of four independent experiments. The results are expressed as the mean ± SD. Induction of apoptosis by cytostatics and demethylating agents was significant in comparison with control; astericks denote p < 0.05 for combination of cytostatics and demethylating agents *versus *cytostatics alone.

Treatment of CRC cells with oxaliplatin or 5-FU alone induced apoptosis in ~40% of the cells. The percentage of apoptotic cells increased when cells were coincubated with decytabine: to 60% in the case of oxaliplatin and to ~55% in the case of 5-FU (Fig. 5). Combination of zebularine with oxaliplatine did not increase apoptosis whereas combination with 5-FU increased significantly the number of apoptotic CRC cells.

In addition, induction of apoptosis in the Colo-205 cells induced by combination of cytostatics with decytabine increased levels of mRNA of *CASP3 *gene and pro-caspase 3 and 8 proteins, as compared with the control. The proteolytic cleavage of the poly (ADP-ribose) polymerase (PARP) was augmented following incubation of cells with cytostatics and decytabine (Fig. 4B).

### The changes in mitochondrial membrane potential (*ΔΨ*_*m*_)

**Figure 6:**
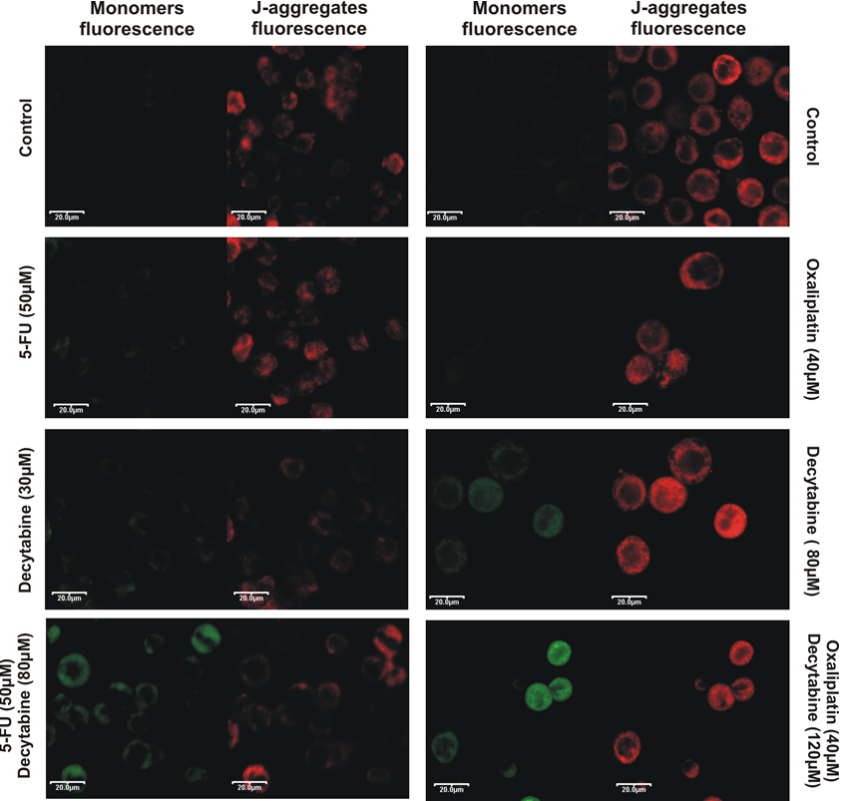
**Microscopic images of changes in mitochondrial membrane potential (Δ*Ψ*_*m*_)**. Cells were incubated with cytostatics, decytabine or their combination for 48 h and examined by confocal microscopy to detect changes in the mitochondrial membrane potential Δ*Ψ*_*m*_. Left image, detection of the monomers (green fluorescence) indicating the presence of depolarized mitochondria (apoptotic cells). Right image, detection of the aggregates (red fluorescence) indicating the functional, polarized mitochondria.

The *ΔΨ*_*m *_was evaluated using potential-sensitive dye MitoLight. Cells, previously treated for 48 h with decytabine, cytostatics or their combinations were loaded with the dye and tested by the confocal microscope. The image analysis revealed high polarization of control cells (Fig. 6). Decytabine in a dose-dependent manner increased membrane depolarization (green fluorescence) and further depolarized cells when coincubated with studied cytostatics. Combinations of decytabine with both cytostatics caused an increase in green with concomitant decrease in red fluorescence signals (Fig. 6)

## Discussion

The important finding of the present work is the demonstration that DNA methyltransferase inhibitor (DNMTi), decytabine, potentiated cytotoxic effects of classical cytostatics, 5-FU and oxaliplatin, in colorectal carcinoma (CRC) cell line Colo-205. The second important finding is that the potentiating effect of decytabine is probably not the class effect of DNMT inhibitors, as the effect of zebularine was qualitatively different.

DNMTi have recently gained attention as agents inhibiting cancer growth including colorectal carcinoma [14]. However, the role of their therapeutic potential could be properly evaluated only in combination with classical agents used in the treatment of CRC, since all new therapeutic agents are introduced as add-on options. Since 5-fluorouracil (5-FU) and oxaliplatin constitute the backbone treatment of CRC [15], we investigated the effects of addition of decytabine or zebularine, methyltransferase inhibitors (DNMTi), to oxaliplatin or 5-FU on survival of Colo-205 human CRC cells *in vitro*.

We have found that studied agents, when given alone, induced the inhibition of Colo-205 cancer cells growth *in vitro *with comparable order of potency (estimated on the basis of survived cells percent), with the exception of zebularine that in low concentrations stimulated growth of CRC cells and was inhibitory only above 45 μM (Fig. 1A and 1B). When cancer cells were treated with the combination of agents, the addition of decytabine to oxaliplatin or 5-FU increased inhibition of growth of cancer cells (Fig. 1A and 1B). The isobolograms, constructed on the basis of IC_50 _values, indicated the synergistic interaction between decytabine and both studied cytostatics, oxaliplatin and 5-FU, and the antagonism between zebularine and oxaliplatin (Fig. 2) and lack of any interaction between zebularine and 5-FU, as only additive effect was observed.

Highly synergistic interaction resulting from addition of decytabine to oxaliplatin and, to a lesser extent, to 5-FU was quantitatively analyzed according to Chou and Talalay method. The obtained Combination Index (CI) was in the case of oxaliplatin and decytabine between 0.12–0.28 and in case of 5-FU and decytabine between 0.25–0.63 in the broad range, 25% to 75%, of fraction affected (Fa) level (Table 1). These ranges are regarded as "high synergism" or "synergism" according to Peters and co-workers [16].

The observed synergistic interaction between decytabine and oxaliplatin was dose-dependent. The observed antagonism between zebularine and oxaliplatin on CRC cells survival increased with the dose of zebularine. It appears, therefore, that two methyltransferase inhibitors, belonging to the same class of agents, influence the inhibitory effects of cytostatics on CRC cells growth in a qualitatively different manner. These differences could be related to the direct effects of studied DNMTis on survival of CRC cells since decytabine by itself induced inhibition of CRC cell growth, whereas zebularine induced non-significant but apparent stimulation of growth of CRC cells and inhibition only at higher concentrations (Fig. 1A and 1B). The interaction between decytabine and both cytostatics was paralleled by significant induction of apoptosis which was higher in case of combination.

The strong synergism between decytabine and cytostatics reported here could be explained by the ability of decytabine to bind covalently with DNMT thus obstructing DNA synthesis and in this way leading to the cell death. Decytabine may also induce DNA damage through structural instability at the site of incorporation [17,18] with the resulting activation of ATM kinase and consequently p53 protein [19], what was confirmed in the present study (Fig. 4).

The most powerful combination, decytabine and oxaliplatin, led to CRC cell cycle arrest in the S phase and, when compared to the control cycle, strong reduction of cell number in the G1 phase (Fig. 3). This was accompanied by increased mRNA level of *ATM*, suggesting induction of ATM-dependent pathway. Activation of this pathway may lead to activation of Chk2 kinase which *via *phosphorylation of Cdc25A induces its degradation. The down regulation of Cdc25A leads to inhibition of DNA synthesis and cell cycle arrest of CRC cells in the S phase, a predominant effect of decytabine and oxaliplatin combination (Fig. 3, 4). Moreover, the synergistic interaction between decytabine and cytostatics led to augmentation of pro-caspase 3 and 8 levels in cytosolic fractions and mRNA level of *p53 *gene (Fig. 4). Thus, the cytotoxic effect of decytabine and cytostatics, could result from apoptosis induced by internal and external signals. Indeed, the possible involvement of mitochondrial pathway was indicated by the finding that combination of decytabine with cytostatics induced the disruption of *ΔΨ*_*m *_(Fig. 6). The combination of decytabine and both cytostatics resulted in powerful apoptotic signal that was manifested by fragmentation of PARP protein into 84 kDa and 25 kDa fragments also indicating apoptosis (Fig. 4).

In summary, we have demonstrated, for the first time, that decytabine, DNMT inhibitor, exerted powerful synergistic interaction with oxaliplatin and 5-FU, inhibiting the survival of CRC cells *in vitro*. In contrast, the influence of other DNMTi, zebularine, on the inhibitory effect of oxaliplatin on survival of CRC cells was antagonistic. Thus, the synergistic interaction of decytabine with cytostatics representing the backbone treatment of CRC is probably not the class effect of DNMTi.

The mechanism of synergism between decytabine and oxaliplatine or 5-FU is probably related to the augmentation of apoptotic signal when compared with either agent applied alone. Such augmentation may suggest that cytotoxic effects of classical cytostatics used in the treatment of colorectal carcinoma, when combined with decytabine, could be obtained at lower doses and with higher probability to induce death of cancer cells. That is why the presented results could be of interest for biologists and clinicians.

## Abbreviations

CI: combination index; DNMTi: DNA methyltransferase inhibitor; CRC: colorectal cancer; DRI: dose reduction index; IC: inhibitory concentration; 5-FU: 5-fluorouracil; Fa: fraction affected; MTT: [3-(4,5-dimethylthiazol-2-yl)-2,5-diphenyl tetrazolium bromide]; PI: propidine iodide.

## Competing interests

The authors declare that they have no competing interests.

## Authors' contributions

SF conceived of the study, designed, coordinated the study, carried out the: proliferation, RT-PCR and flow cytometry assays, interaction and statistical analysis and drafted the manuscript; AG carried out the proliferation and western blotting assays; IM worked as an confocal microscopy specialist for the examination of the samples; JS revised the manuscript critically for important intellectual content and helped draft the manuscript.
